# Pamidronate therapy for hypercalcemia and congenital mesoblastic nephroma: a case report

**DOI:** 10.1186/1757-1626-2-9315

**Published:** 2009-12-12

**Authors:** Fahimeh Soheilipour, Mahmood Ashrafi Amineh, Mahin Hashemipour, Ali Asghar Salahi Kojoor, Amir Hosein Davarpanah Jazi

**Affiliations:** 1 Department of Pediatric Endocrinology, Isfahan University of Medical Sciences, Isfahan, Iran; 2 Department of Pediatric Surgery, Isfahan University of Medical Sciences, Isfahan, Iran; 3 Medical Education Research Center, Isfahan University of Medical Sciences, Isfahan, Iran

## Abstract

Hypercalcemia can causes life threatening complications. We report an infant with severe hypercalcemia due to congenital mesoblastic nephroma. Hypercalcemia was corrected before nephrectomy by pamidronate. According to our knowledge this is a rare case with severe neoplasm induced hypercalcemia among neonates who treated by bisphosphonates. The aim of this report is to define new approach to neoplasm induced neonatal hypercalcemia.

## Introduction

In infants, hypercalcemia is a rare but serious condition. Some causes of hypercalcemia in neonatal period include severe primary hyperparathyroidism, homozygous familial hypocalciuric hypercalcemia, idiopathic infantile hypercalcemia, William's syndrome, iatrogenic administration of calcium (generally intravenously), vitamin A or D intoxication and subcutaneous fat necrosis or as a form of paraneoplastic syndromes. They can be complicated by potentially life-threatening hypercalcemia which should be investigated and treated without delay [1,2].

Life-threatening hypercalcemia is rare in infants and young children. Pharmacological treatment of severe hypercalcemia is complicated by lack of experience with some effective medications such as bisphosphonates in newborns.

Congenital mesoblastic nephroma is the most common renal tumor in neonatal and early infantile period. It can be associated with paraneoplastic syndromes, such as hypertension and hypercalcemia [1].

We report a 9 day old newborn infant with history of polyhydramnios, a right-sided renal mass duo to mesoblastic nephroma, hypercalcemia and hypertension which was treated with a bisphosphonate, pamidronate, to stabilize life-threatening hypercalcemia before nephrectomy.

## Case description

A 9-day old male newborn infant was admitted in our hospital with history of poor feeding, vomiting, decreased tonicity, lethargy, and dehydration since two days before. He was the second-born child of non consanguineous parents. His mother had history of polyhydramnios. He was the product of caesarian section because of polyhydramnios. Birth weight was 3700 g, Apgar scores were normal and he was discharged at the first day of age.

On admission findings were as follows: heart rate 143/min, respiratory rate 52/min, blood pressure (BP) 140/110 mmHg, weight 2.30 kg. He had lost 1400 g since birth and had a mild degree of hypotonia and hyporeflexia.

**Figure 1:**
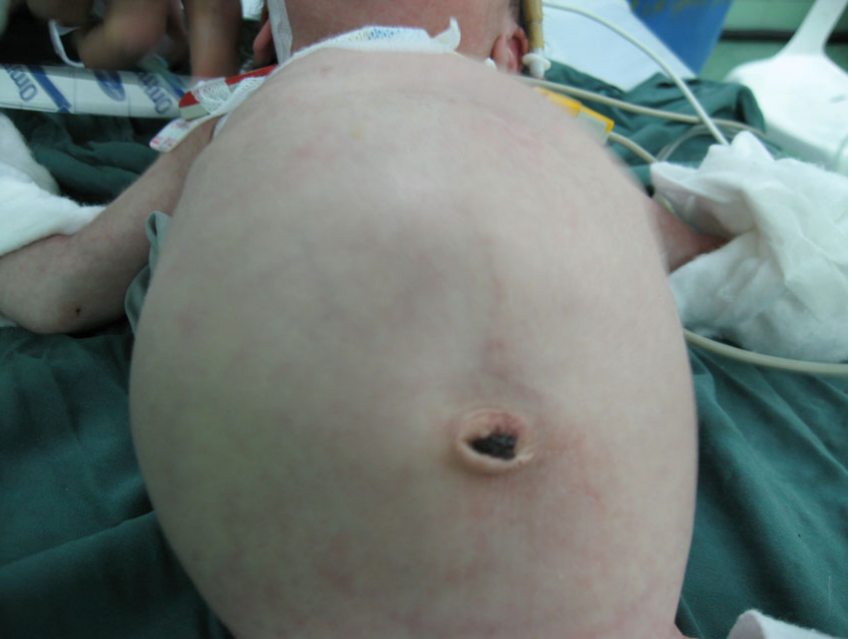
**The figure shows the huge abdominal mass dominant in right side**.

Physical examination revealed a large right-sided abdominal mass with regular margins (Fig 1). Ultrasound examination showed a large echogenic and heterogeneous mass composed cystic areas that was 85 × 65 mm confirmed.

Initial biochemical studies revealed serum calcium of 17 mg/dl (reference range 8.2-10.5 mg/dl). He had persistent hypercalcemia (serum calcium > 15 mg/dl) for more than 6 hours before medical therapy. Other laboratory tests were in normal ranges and serum Phosphorus and magnesium were 3.8 mg/dl and 2 mg/dl respectively.

At first visit we tried normal saline and furosemide but no obvious response was seen. Then the patient received two intravenous pamidronate (1.5 mg/kg) for 2 days. Pamidronate infused in 25 milliliter of 5% dextrose saline solution over 4 hours. His serum calcium level decreased significantly, and about 18 hours later, his total calcium level normalized and his symptoms recovered except abdominal mass. Because our patient was hypertensive, we prescribed nifedipine also. Unfortunately we do not have the levels of serum PTH and specific markers of bone turnover because were not measured.

After stabilization of hypercalcemia, successful right nephrectomy was performed.

**Figure 2:**
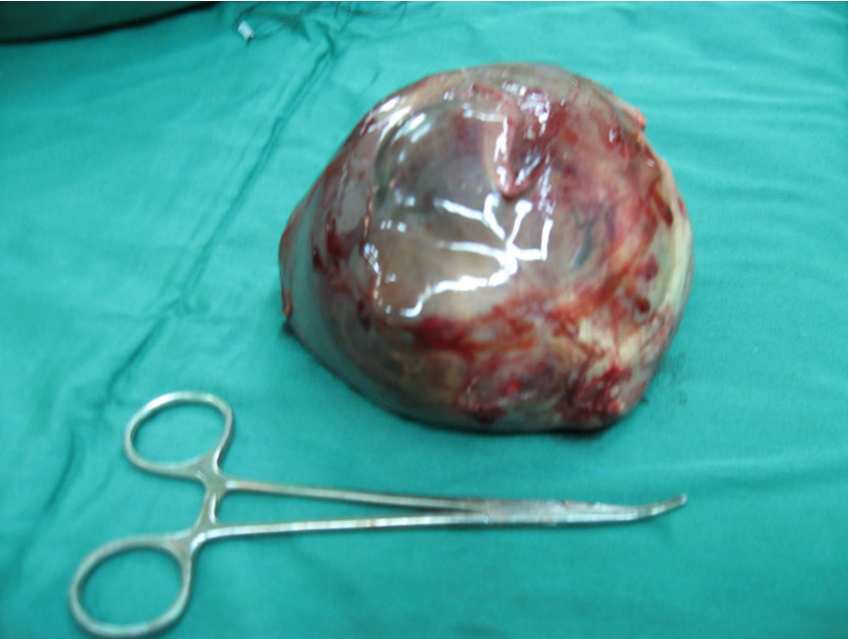
**Gross picture of removed mass**.

**Figure 3:**
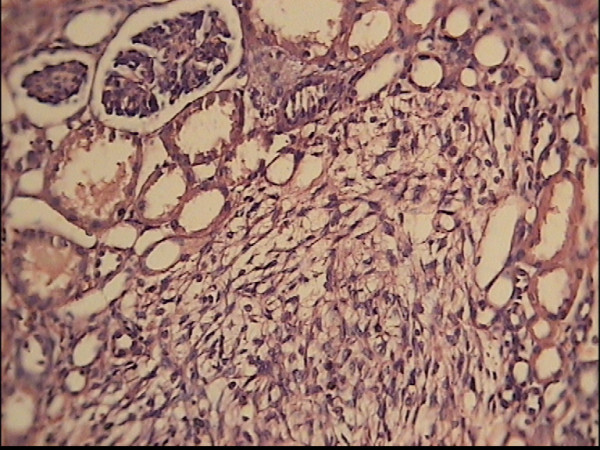
**Hematoxiline and eosin staining of the tumor specimen showed bundles of spindle shaped stromal cells with occasional entrapped normal renal tubular and glomerular cell**. (1000×).

Histological evaluation of revealed a well circumscribed, 11 × 10 × 8 cm, round shape, smooth yellow mass in the upper pole of the right kidney (Fig 2). On the microscopic examination the lesion displayed bundles of spindle shaped stromal cells with occasional entrapped normal renal tubular and glomerular cell, findings that consistent with diagnosis of mesoblastic nephroma (Fig 3). After operation serum calcium level doesn't increase again. The normal blood pressure achieved about 16 hours after operation.

## Discussion

We described a neonate with severe paraneoplastic hypercalcemia. Hypercalcemia in this situations, have been reported to be caused by the tumor secretion of PTH, PTH-related peptide [2], prostaglandin E2 [3] and glucagon-like peptides [4]. When the total calcium concentration is 14 mg/dL, emergency intervention is necessary because of life-threatening adverse effects of hypercalcemia on heart, brain, kidney, and gastrointestinal function. Hypercalcemia that is an uncommon complication of childhood cancers, has been reported in association with childhood renal tumors in 1.2% of cases, compared with 0.7% among childhood solid tumors in general [5,6].

Despite the response to pharmacological therapy with hydration and furosemide diuresis, in many times is inadequate and transient, pharmacological treatment of hypercalcemia with medications such as bisphosphonates is complicated by lack of experience in newborns. We use pamidronate, to stabilize hypercalcemia, prior to nephrectomy. The serum calcium was successfully decreased without any complications.

Fox et al for the first time described the use of pamidronate to control marked hypercalcemia in neonatal hyperparathyroidism that resulted from an inactivating mutation of the calcium-sensing receptor. They treated their neonate, with intravenous pamidronate (20 mg/m^2^). Six doses of 20 to 30 mg/m^2 ^were given over the next 6 weeks with the aim of performing parathyroidectomy once it was technically feasible and she was clinically stable [7]. Although total dose of pamidronate in this case was higher than our patients but each prescribed pamidronate doses was lower. It may due to lower calcium level in their patient.

In another study by Alos et al, Four newborns presented with subcutaneous fat necrosis complicated by severe hypercalcemia were reported. Despite traditional treatment, calcium levels persistently remained high. By using 3-4 doses of pamidronate (0.25-0.50 mg/kg/dose) calcium levels decreased within 48-96 h [8].

Bryowsky et al reported a 17-day-old premature infant who received 0.7 mg/kg of pamidronate for treatment of hypercalcemia due to parenteral nutrition. His serum calcium concentration returned to normal without any adverse reaction. They also propose clinical trials in pediatric patients are necessary to determine how best to use bisphosphonates in these patient populations [9].

Despite a few studies like these, there are a few controlled studies that have been performed on the use of pamidronate, to stabilize life-threatening hypercalcemia among newborns; therefore, dosing guidelines are not available. Some mentions doses for intravenous pamidronate administration in newborn studies for treatment of hypercalcemia resulting from different causes have varied from 0.35 to 1.2 mg/kg per dose or 0.25-0.50 mg/kg/dose [8]. In our case higher dose of tolerable pamidronate was administered and therefore rapid response to treatment was achieved in such critical emergent setting.

## Conclusion

Intravenous pamidronate appears to be a safe and effective treatment for severe hypercalcemia among neonates and infants with life-threatening paraneoplastic hypercalcemia. This could stabilize the patients before surgery especially those who do not respond adequately to traditional treatments or when urgent surgery is impassible.

## Consent

Written informed consent was obtained from the patient for publication of this case report and accompanying images. A copy of the written consent is available for review by the Editor-in-Chief of this journal.

## Competing interests

The authors declare that they have no competing interests.

## Authors' contributions

FS and MH analyzed and interpreted the patient data as well as make the proper diagnosis. MAA and AASK performed the surgical procedure. FS and AHDJ were the major contributors in writing the manuscript. All authors read and approved the final manuscript.
